# Implications of insecticide resistance for malaria vector control with long-lasting insecticidal nets: a WHO-coordinated, prospective, international, observational cohort study

**DOI:** 10.1016/S1473-3099(18)30172-5

**Published:** 2018-06

**Authors:** Immo Kleinschmidt, John Bradley, Tessa Bellamy Knox, Abraham Peter Mnzava, Hmooda Toto Kafy, Charles Mbogo, Bashir Adam Ismail, Jude D Bigoga, Alioun Adechoubou, Kamaraju Raghavendra, Jackie Cook, Elfatih M Malik, Zinga José Nkuni, Michael Macdonald, Nabie Bayoh, Eric Ochomo, Etienne Fondjo, Herman Parfait Awono-Ambene, Josiane Etang, Martin Akogbeto, Rajendra M Bhatt, Mehul Kumar Chourasia, Dipak K Swain, Teresa Kinyari, Krishanthi Subramaniam, Achille Massougbodji, Mariam Okê-Sopoh, Aurore Ogouyemi-Hounto, Celestin Kouambeng, Mujahid Sheikhedin Abdin, Philippa West, Khalid Elmardi, Sylvie Cornelie, Vincent Corbel, Neena Valecha, Evan Mathenge, Luna Kamau, Jonathan Lines, Martin James Donnelly

**Affiliations:** a MRC Tropical Epidemiology Group, Department of Infectious Disease Epidemiology, London School of Hygiene & Tropical Medicine, London, UK; b Department of Disease Control, London School of Hygiene & Tropical Medicine, London, UK; c School of Public Health, University of the Witwatersrand, Johannesburg, South Africa; d Global Malaria Programme, WHO, Geneva, Switzerland; e Federal Ministry of Health, Khartoum, Sudan; f School of Biological Sciences, Universiti Sains Malaysia, Penang, Malaysia; g KEMRI Centre for Geographic Medicine Research Coast, Kilifi, Kenya; h Khartoum Malaria Free Initiative, Khartoum, Sudan; i National Reference Unit (NRU) for Vector Control, The Biotechnology Center, University of Yaoundé I, Yaoundé, Cameroon; j Programme National de Lutte contre le Paludisme (PNLP), Ministère de la Santé, Cotonou, Benin; k National Institute of Malaria Research, Indian Council of Medical Research, Department of Health Research, New Delhi, India; l University of Khartoum, Faculty of Medicine, Department of Community Medicine, Khartoum, Sudan; m KEMRI/CDC Research and Public Health Collaboration, Kisumu, Kenya; n National Malaria Control Program, Ministry of Public Health, Yaoundé, Cameroon; o Organisation de Coordination pour la lutte contre les Endemies en Afrique Centrale (OCEAC), Yaoundé, Cameroon; p Faculty of Medicine and Pharmaceutical Sciences, University of Douala, Douala, Cameroon; q Centre de Recherche Entomologique de Cotonou, Cotonou, Benin; r University of Nairobi, School of Medicine, College of Health Sciences, Department of Medical Physiology, Nairobi, Kenya; s Department of Vector Biology, Liverpool School of Tropical Medicine, Liverpool, UK; t Faculté des Sciences de la Santé, Université d'Abomey-Calavi, Cotonou, Benin; u Maladies Infectieuses et Vecteurs, Ecologie, Génétique, Evolution et Contrôle (MIVEGEC), Institut de Recherche pour le Développement (IRD), CNRS, University of Montpellier, Montpellier, France; v KEMRI Eastern and Southern Africa Centre of International Parasite Control, Nairobi, Kenya; w KEMRI Centre for Biotechnology and Research Development, Nairobi, Kenya; x Malaria Programme, Wellcome Trust Sanger Institute, Hinxton, Cambridge, UK

## Abstract

**Background:**

Scale-up of insecticide-based interventions has averted more than 500 million malaria cases since 2000. Increasing insecticide resistance could herald a rebound in disease and mortality. We aimed to investigate whether insecticide resistance was associated with loss of effectiveness of long-lasting insecticidal nets and increased malaria disease burden.

**Methods:**

This WHO-coordinated, prospective, observational cohort study was done at 279 clusters (villages or groups of villages in which phenotypic resistance was measurable) in Benin, Cameroon, India, Kenya, and Sudan. Pyrethroid long-lasting insecticidal nets were the principal form of malaria vector control in all study areas; in Sudan this approach was supplemented by indoor residual spraying. Cohorts of children from randomly selected households in each cluster were recruited and followed up by community health workers to measure incidence of clinical malaria and prevalence of infection. Mosquitoes were assessed for susceptibility to pyrethroids using the standard WHO bioassay test. Country-specific results were combined using meta-analysis.

**Findings:**

Between June 2, 2012, and Nov 4, 2016, 40 000 children were enrolled and assessed for clinical incidence during 1·4 million follow-up visits. 80 000 mosquitoes were assessed for insecticide resistance. Long-lasting insecticidal net users had lower infection prevalence (adjusted odds ratio [OR] 0·63, 95% CI 0·51–0·78) and disease incidence (adjusted rate ratio [RR] 0·62, 0·41–0·94) than did non-users across a range of resistance levels. We found no evidence of an association between insecticide resistance and infection prevalence (adjusted OR 0·86, 0·70–1·06) or incidence (adjusted RR 0·89, 0·72–1·10). Users of nets, although significantly better protected than non-users, were nevertheless subject to high malaria infection risk (ranging from an average incidence in net users of 0·023, [95% CI 0·016–0·033] per person-year in India, to 0·80 [0·65–0·97] per person year in Kenya; and an average infection prevalence in net users of 0·8% [0·5–1·3] in India to an average infection prevalence of 50·8% [43·4–58·2] in Benin).

**Interpretation:**

Irrespective of resistance, populations in malaria endemic areas should continue to use long-lasting insecticidal nets to reduce their risk of infection. As nets provide only partial protection, the development of additional vector control tools should be prioritised to reduce the unacceptably high malaria burden.

**Funding:**

Bill & Melinda Gates Foundation, UK Medical Research Council, and UK Department for International Development.

## Introduction

Deployment of insecticide-based interventions has been the principal driver of reductions in the global malaria burden since 2000. The massive scale-up of insecticide-treated nets resulted in more than 50% of people in malaria endemic areas in sub-Saharan Africa sleeping under nets in 2016.^1^ Of 663 million clinical malaria cases averted in sub-Saharan Africa since 2001, 78% were averted thanks to the use of insecticide-treated nets and indoor residual spraying.^2^ Any loss of effectiveness of these interventions could therefore cause a catastrophic rebound in disease incidence and mortality.

Resistance to insecticides is widespread in anopheles mosquitoes in sub-Saharan Africa and India, especially resistance to pyrethroids, the class of insecticide used on all long-lasting insecticidal nets. Pyrethroids are increasingly less effective at killing mosquitoes^3^, ^4^ and mathematical models predict this drop in effectiveness could lead to increased malaria incidence.^5^ However, little evidence has been reported of an epidemiological effect resulting from resistance. An often cited example of insecticide resistance leading to control failure is KwaZulu-Natal, South Africa in the late 1990s, where despite good indoor residual spraying coverage, a ten-times increase in malaria cases was reversed when pyrethroid spraying was replaced with dichloro-diphenyltrichloroethane (DDT) spraying in response to reported pyrethroid resistance in a local malaria vector, *Anopheles funestus*.^6^ This apparent control failure was with indoor residual spraying, not insecticide-treated nets, and could have been confounded by the simultaneous introduction of artemether plus lumefantrine as a first-line treatment following reported resistance to sulfadoxine plus pyrimethamine. To date, no convincing examples of long-lasting insecticidal net malaria control failure due to pyrethroid resistance have been reported. Studies from Malawi^7^ and Kenya^8^ showed that insecticide-treated nets protected against malaria infection in areas with substantial amounts of pyrethroid resistance.

**Research in context**

**Evidence before this study**

Nearly 70% of the more than 660 million clinical cases of malaria that were averted between 2000 and 2015, have been attributed to the use of insecticide-treated nets. Therefore, to quantify the likely effects of emergent insecticide resistance on disease burden is a major public health imperative. We searched the PubMed database, with no date or language restrictions, using the terms “insecticide resistance” or “pyrethroid resistance”, “malaria”, “effectiveness” or “efficacy”, and “long lasting insecticidal net (LLIN)” or “insecticide treated net (ITN)”. We found a predominance of entomological or laboratory studies, several mathematical modelling studies, and a small number of studies describing the effect on epidemiological outcomes. All studies describing the effect on epidemiological outcomes were observational. Some studies compared malaria incidence or prevalence before and after long-lasting insecticidal net distribution, and others evaluated the effectiveness of long-lasting insecticidal nets in the presence of insecticide resistance in a single location, either through cross-sectional surveys or cohort follow-up. None of these studies had simultaneous characterisation of disease or infection burden and insecticide resistance. Entomological studies generally concluded that reduced mosquito mortality in bioassay tests and experimental hut studies would affect malaria control, but without evidence of an effect in humans. Epidemiological studies either found that net users were at least partly protected against malaria compared with non-users, even in the presence of resistance, or found that the effect on malaria after universal long-lasting insecticidal net distribution was suboptimal, thus concluding that this was due to insecticide resistance.

**Added value of this study**

In a WHO-coordinated initiative, we measured pyrethroid resistance and the effectiveness of long-lasting insecticidal nets concurrently in prospective studies (cohort and cross-sectional) in 279 locations in five countries. We compared effectiveness of long-lasting insecticidal nets at locations with differing levels of insecticide resistance. We showed that resistance is highly variable in time and space. We found that nets provided protection against malaria in most locations, irrespective of the presence of insecticide resistance. Previous studies did not compare effectiveness at locations that differed in resistance, and did not assess the effect of resistance on malaria burden in as many locations and settings as we did in this study.

**Implications of all the available evidence**

Evidence suggests that using a long-lasting insecticidal net provides protection against malaria, even in areas with pyrethroid resistance. Whether pyrethroid only long-lasting insecticidal nets are as effective as they were before the onset of resistance, and whether long-lasting insecticidal nets might become less effective in areas with higher amounts of resistance than were encountered in our study, is not known. Universal access to long-lasting insecticidal nets, together with campaigns to achieve greater use of nets, should continue in malaria endemic areas, even in the presence of insecticide resistance. However, in some locations malaria incidence remained high despite high use of nets, emphasising the need for new tools and approaches for malaria prevention if targets for the reduction of the global malaria burden are to be achieved, and to forestall a potential rebound of malaria due to higher resistance in the future.

In 2012, WHO released the Global Plan for Insecticide Resistance Management in Malaria Vectors^9^ to slow the development of insecticide resistance. This plan has been a challenge for national programmes to implement because of its primary focus on switching chemical classes for indoor residual spraying.^4^, ^10^

This multicountry prospective study was coordinated by WHO to assess the effect of insecticide resistance on malaria disease burden and on the performance of long-lasting insecticidal nets.^11^ We aimed to address two questions. First, are long-lasting insecticidal nets protective against malaria in the presence of vector resistance to pyrethroids, and second, are higher frequencies of vector resistance to pyrethroids associated with greater infection prevalence and incidence of clinical malaria at the community level? A third aim, to assess the effect on insecticide resistance of scaling-up insecticide-based interventions, will be addressed in a separate paper.

## Methods

### Study design and participants

This prospective, observational cohort study was done at 279 distinct geographical locations (subsequently referred to as clusters) in five countries (Benin, Cameroon, India, Kenya, and Sudan), representing a range of pyrethroid resistance and malaria transmission scenarios. Each cluster was a village or group of villages in which phenotypic resistance could be measured. The study was observational, as resistance cannot be randomly assigned, and because it is unethical to randomly allocate long-lasting insecticidal nets to study populations in malaria endemic areas.^11^

Insecticide resistance and malaria prevalence and incidence were measured over consecutive years, alongside each other in all study clusters to increase statistical power and capture variation in disease burden and effectiveness of nets in relation to variation in insecticide resistance across a wide range of locations and over time.

In each cluster, cohorts of children from randomly selected households were recruited and followed up by community health workers to measure clinical malaria incidence. The same cohort was followed over several years in four countries (Kenya: September, 2013 to December, 2015; India: May, 2015, to November, 2016; Sudan: June, 2012, to December, 2014; and Benin: February, 2013, to December, 2014, with a separate cohort from May to November, 2015), whereas in Cameroon cohorts were recruited for each malaria season from September to December each year from 2013 to 2015. In high transmission sites (Benin, Cameroon, and Kenya), children aged 6 months to 5 years were eligible for inclusion, in India children aged 6 months to 14 years were eligible, and in Sudan children aged 6 months to 10 years were eligible. We used no other eligibility criteria, except that the child should be normally resident in the cluster.

Pyrethroid long-lasting insecticidal nets were the principal form of malaria vector control in all study areas; in Sudan this approach was supplemented by indoor residual spraying.^12^ Long-lasting insecticidal net effectiveness was assessed by comparing malaria incidence as rate ratios (RR) and prevalence as odds ratios (OR) between net users and non-users. Long-lasting insecticidal net effectiveness was compared at locations with differing amounts of resistance. A standardised study framework was applied across countries,^11^ but there were minor amendments to account for differences in local policy and practice.

Ethical approval was obtained from national ethics committees (approval numbers 102/CNE/SE/09 [Cameroon], 116-12-09 [Sudan], SSC/ERC No. 1677 [Kenya], ECR/NIMR/EC/2010/75 [India], and 007 [25 May 2010] [Benin]). Participation in each component of the study was subject to written informed consent by the parent or guardian, and additional assent by children aged 10 years and older.

### Procedures

Mosquitoes were collected seasonally in each cluster to assess susceptibility to pyrethroids. Larvae were reared until they were 2–5-day-old adults, and female mosquitoes were exposed to the discriminating dose of deltamethrin using the standard WHO bioassay test; in India it was necessary to use mixed age, wild-caught mosquitoes or F1 progeny for susceptibility testing.^13^ Mortality was recorded 24 h after deltamethrin exposure. Cluster and year specific mosquito mortality measurements were dichotomised as either high or low resistance in relation to the median mortality of all measurements from all study clusters.

For vector control, long-lasting insecticidal net distributions provided coverage of one net per two people across all five study sites. Nets were distributed in Benin in 2011 (Olyset Net; Sumitomo Chemical, Tokyo, Japan; 1 g/m^2^ permethrin) and 2014 (PermaNet 2.0; Vestergaard, Lausanne, Switzerland; 55 mg/m^2^ deltamethrin), in Cameroon in 2011 and 2015 (PermaNet 2.0), in India in 2014 (PermaNet 2.0), in Kenya in 2012 and 2014 (Olyset Net), and in Sudan in 2011 and 2014 (PermaNet 2.0). In Sudan, half of the clusters were randomised to receive two rounds of indoor residual spraying with bendiocarb (Ficam 80% wettable powder; Bayer, Leverkusen, Germany; 200 mg active ingredient per m^2^) each year, except for a small number of indoor residual spraying clusters that were sprayed with two rounds of deltamethrin (Chema Industries, Alexandria, Egypt; 25 mg active ingredient per m^2^) in 2012.^12^

For active case detection, each child was visited regularly (fortnightly in Cameroon, Sudan, and India, and monthly in Benin and Kenya). Those with an axillary temperature greater than 37·5°C or reporting fever in the previous 2 weeks were tested using a rapid diagnostic test (in Cameroon, India, Kenya, and Sudan; CareStart Malaria HRP2 [Pf]; Access Bio, Somerset, NJ, USA [used in Kenya and Cameroon]; SD Bioline Malaria Ag P.f/Pan; Standard Diagnostics, Gyeonggi-do, South Korea [used in Kenya and Sudan]; and SD Bioline Malaria, Alere Medical, Gurgaon, India [used in India]) or microscopy (in Benin). Children who tested positive were treated according to national guidelines or were referred to a health facility. If caregivers reported upon questioning that a child had visited a health facility since the last visit, the community health workers recorded confirmed malaria diagnoses from health facility records. An adult caregiver was asked whether the child slept under a long-lasting insecticidal net the night before each visit, except in Cameroon where net use was an inclusion criterion. Children who reached the upper age limit of eligibility were excluded from further assessments and replaced by a younger child, usually a sibling from the same household.

Cross-sectional surveys were done to measure infection prevalence during the transmission season (Benin in July, 2015; Cameroon in October, 2013, and October, 2014; Kenya in December, 2012, and December, 2014; India in August, 2015, and June and November, 2016; and Sudan in September of 2012, 2013, and 2014). In Benin, Cameroon, and Kenya, households were randomly selected from lists prepared for each cluster. In Sudan, a random subset of the cohort was selected, whereas in India the entire cohort was included. Participating children were tested for malaria parasites and the caregiver was asked whether the child slept under a long-lasting insecticidal net the night before the survey.

Slides were read by two trained microscopists for results in both cohort and cross-sectional studies. Discordance was settled by a third reader.

### Statistical analysis

Sample size was determined separately for each country^11^ to provide 80% power to detect minimum differences in clinical incidence ranging from 30% (Sudan) to 54% (Benin) between high and low resistance clusters. When the results of the five countries were combined, there was 80% power to detect a 15% difference in malaria incidence between high and low resistance clusters, assuming a coefficient of variation between clusters of 0·45.

Malaria incidence was estimated as the number of incident cases per child-year of follow-up. Consecutive positive test results were counted as one episode because the second positive test could be a false positive from rapid detection tests that detect retained parasite antigen from an already cleared infection. A child was considered not at risk for a period of 2 weeks after a positive test result because of the prophylactic effect of treatment. Follow-up was censored for periods when a child was absent. Each year's follow-up was linked with the mosquito mortality measurement taken in that cluster for that year. For cohorts that continued for more than 1 year, follow-up was split into calendar years. For Benin in 2013, where bioassays were done in June, insecticide resistance measurements were assigned to follow-up from January to July, and for bioassays done in October, insecticide resistance measurements were assigned to follow-up from August to December. For India, where mosquito mortality was measured in December, 2015, resistance measurements were related to follow-up data from May, 2015, to June, 2016, and for mosquito mortality measurements in December, 2016, resistance measurements were related to follow-up data from July, 2016, to December, 2016. We used RRs to compare incidence during follow-up stratified by reported long-lasting insecticidal net use. We compared incidence RRs for long-lasting insecticidal net use versus non-use between areas of high and low resistance (interaction test) using Poisson regression adjusted for age, district, calendar month, and in the case of Sudan, indoor residual spraying. We used random effects meta-analysis to compute the overall RRs of effectiveness of long-lasting insecticidal nets for high and low resistance strata, for all countries combined, and separately for all countries, excluding Sudan. Results were displayed as forest plots. Results are presented with and without Sudanese data because it was the only country in which indoor residual spraying was used in addition to long-lasting insecticidal nets, and where net use was positively associated with malaria incidence and prevalence.

In separate Poisson regression models, we investigated the effect of insecticide resistance on case incidence. We used RRs to compare high and low insecticide resistance clusters, dichotomised as either high or low resistance in relation to the median mortality of all measurements from all study clusters. The incidence RR per 10% decrease in mosquito mortality was calculated similarly using resistance as a continuous variable. RRs and interaction tests were adjusted for age, district, long-lasting insecticidal net use, and calendar month, and indoor residual spraying in the case of Sudan.

Prevalence of infection was calculated as the number of people who tested positively divided by the total number tested in cross-sectional surveys. Analyses analogous to those described above were done for cross-sectional prevalence data using logistic regression to estimate ORs, comparing prevalence of infection between those reporting long-lasting insecticidal net use the night before the survey, and those reporting not to have used a net. We compared ORs for net use versus non-use between areas of high and low resistance using logistic regression adjusted for age, district, and in the case of Sudan, indoor residual spraying. We used random effects meta analysis to compute the overall ORs of effectiveness of long-lasting insecticidal nets for high and low resistance strata for all countries combined, and separately for all countries, excluding Sudan.

In separate logistic regression models, we investigated the effect of insecticide resistance on infection prevalence. We used ORs to compare high and low resistance clusters, dichotimised in relation to the median mortality of all measurements from all study clusters. The OR per 10% decrease in mosquito mortality was calculated using resistance as a continuous variable.

We produced cluster level standardised prevalence residuals by subtracting the survey mean and dividing by the survey SD. Cluster level standardised incidence residuals were calculated similarly. We created scatter plots of standardised prevalence and incidence against mosquito mortality.

In India, a socioeconomic status variable was calculated for each study participant, based on occupation, education, and income of the head of household.^14^ Socioeconomic status was included in separate models of incidence and prevalence and net use for India to investigate whether this variable confounded the relationship between malaria and net use.

In all statistical models, we used generalised estimating equations^15^ and robust standard errors to account for intracluster correlation of responses (appendix, pp 1–2). Statistical analysis was done using Stata 14.2.

### Role of the funding source

The funder had no role in the study design, data collection, data analysis, data interpretation, or writing of the report. The corresponding author had full access to the study data and had final responsibility for the decision to submit for publication.

## Results

Between June 2, 2012, and Nov 4, 2016, 40 251 children were enrolled and assessed for clinical incidence during 1·4 million follow-up visits. About 65 000 mosquitoes were assessed for insecticide resistance. 681 bioassay measurements were done between Aug 9, 2011, and Sept 16, 2016, at the 279 distinct geographical clusters.

Details of study settings, predominant vector species, malaria endemicity, and long-lasting insecticidal net use in each country were summarised (table 1; appendix p 3). Median cluster-level bioassay mortality was 78·6% (IQR 58·0–92·0), with considerable variation between clusters, countries, and years (table 2). In 78% of bioassay measurements mortality was less than 90%.

**Table 1:** Study setting characteristics by country

	**Benin**	**Cameroon**	**Kenya**	**India**	**Sudan**
Study locations	Districts of Ifangni, Sakété, Pobé, and Kétou (Departement de Plateau)	Districts of Garoua, Pitoa, and Mayo Oulo (north region)	Districts of Teso, Rachuonyo, Nyando, and Bondo (western Kenya)	Subdistrict of Keshkal (Kondagaon, Chhattisgarh)	El Hoosh and Hag Abdalla (Gezira state); Galabat (Gedarif state); New Halfa (Kassala state)
Study clusters	32	38	50	80	79
Predominant malaria vectors	*Anopheles gambiae* subspecies and *Anopheles colluzzii*	*Anopheles arabiensis, A gambiae* subspecies, and *Anopheles funestus*	*A gambiae* subspecies*, A arabiensis*, and *A funestus*	*Anopheles culicifacies*	*A arabiensis*
Vector control interventions	High coverage of insecticide-treated nets (primarily PermaNet 2.0) in all clusters	High coverage of insecticide-treated nets (PermaNet 2.0) in all clusters	High coverage of insecticide-treated nets (PermaNet 2.0 and Olyset Net) in all clusters; Rachuonyo and Nyando received indoor residual spraying with deltamethrin and λ-cyhalothrin in 2012, but no indoor residual spraying was done subsequently	High coverage of insecticide-treated nets (PermaNet 2.0) in all clusters	High coverage of insecticide-treated nets (PermaNet 2.0) in all study clusters; in each study area, half of clusters were randomly allocated to receive additional indoor residual spraying with bendiocarb, balanced by baseline kdr frequencies
Baseline insecticide resistance (cluster-specific range)	kdr frequency by cluster ranged from 44–93% (2011); WHO bioassay mortality to deltamethrin ranged from 20–100% (2011)	kdr frequency by cluster ranged from 9–65% (2011); WHO bioassay mortality to deltamethrin ranged from 43–100% (2012)	WHO bioassay mortality to deltamethrin ranged from 1–100% (2011)	WHO bioassay mortality to deltamethrin ranged from 86–100% in 2013	kdr frequency by cluster ranged from 8·3–70·8% (2010); WHO bioassay mortality to deltamethrin in sentinel clusters ranged from 47–100% (2011)
PfPR_2–10_ endemicity class*	High	High	High	Low	Low

Unless otherwise indicated there was no contemporaneous indoor residual spraying done in the study areas.

* PfPR_2–10_ is the proportion of children aged 2–10 years in the general population who are infected with *Plasmodium falciparum*, averaged over the 12 months of 2010 as estimated by Malaria Atlas Project (MAP);^16^ low PfPR_2–10_ is 0% to ≤5%, intermediate PfPR_2–10_ is >5% to ≤40%, and high PfPR_2–10_ is >40%. kdr=knockdown resistance.

**Table 2:** Insecticide resistance by year

		**Mosquitoes tested**	Median (range) cluster level mosquito mortality*
**Benin (*Anopheles gambiae* sensu lato)**			
	2013	4756	95% (61–100) [91–99]
	2014	2433	47% (19–73) [38–56]
	2015	1580	55% (19–85) [47–69]
**Cameroon (*Anopheles gambiae* sensu lato)**			
	2013	2248	79% (27–100) [51–90]
	2014	2572	74% (20–99) [52–86]
	2015	2945	62% (25–93) [44–77]
**Kenya (*Anopheles gambiae* sensu lato)**			
	2012	1523	77% (10–100) [65–83]
	2013	3333	87% (56–100) [81–97]
	2014	4896	68% (4–97) [42–81]
	2015	4692	71% (15–100) [46–90]
**Sudan (*Anopheles arabiensis*)**			
	2012	4238	73% (30–100) [61–82]
	2013	6450	80% (43–100) [66–89]
	2014	6080	51% (3–90) [35–67]
**India (*Anopheles culicifacies* sensu lato)**			
	2015	8112	84% (57–100) [73–96]
	2016	8316	96% (77–100) [92–98]
All bioassays combined		64 174	79% (3–100) [58–92]

Predominant vectors are shown in parentheses next to countries.

* IQRs are indicated in square brackets.

wSleeping under a long-lasting insecticidal net was associated with lower clinical malaria incidence (table 3). When Sudan was excluded, the protective effect of nets was greater than the overall estimate. In Sudan, there was weak evidence of long-lasting insecticidal net use being associated with higher malaria incidence (RR 1·33, 95% CI 0·97–1·82). Dichotomising cluster–time instances by median mosquito mortality showed no evidence that the effect of nets differed between high and low resistance clusters, whether Sudan was excluded or included (table 3).

**Table 3:** Incidence of clinical malaria cases

			Mean malaria incidence per person-year (95% CI)*					**Effect of net use and resistance in all five countries combined**†			**Effect of net use and resistance in Benin, Cameroon, Kenya, and India combined**†		
			Benin	Cameroon‡	Kenya	Sudan	India	Total resistance observations	Unadjusted rate ratio (95% CI)	Adjusted§ rate ratio (95% CI)	Total resistance observations	Unadjusted rate ratio (95% CI)	Adjusted§ rate ratio (95% CI)
Overall incidence			0·48 (0·39–0·59) [715/1496]	1·33 (1·11–1·60) [1509/1132]	0·82 (0·68–1·00) [4780/5798]	0·033 (0·024–0·044) [1322/40 583]	0·030 (0·020–0·044) [254/8555]	..	..	..	..	..	..
Caregiver-reported use of net on the previous night													
	Non-net use		0·70 (0·51–0·95) [92/132]	..	1·38 (1·02–1·87) [339/245]	0·024 (0·018–0·033) [384/15 848]	0·090 (0·055–0·147) [89/987]	588	1 (ref)	1 (ref)	397	1 (ref)	1 (ref)
	Net use		0·46 (0·36–0·57) [623/1365]	..	0·80 (0·65–0·97) [4331/5442]	0·038 (0·038–0·052) [938/24 735]	0·023 (0·016–0·033) [165/7319]	588	0·65 (0·42–1·01); p=0·055	0·62 (0·41–0·94); p=0·024	397	0·49 (0·34–0·70); p<0·0001	0·48 (0·33–0·69); p<0·0001
Insecticide resistance													
	Low resistance (mortality ≥78·57%)		0·63 (0·48–0·85) [312/495]	1·21 (0·80–1·85) [530/437]	0·83 (0·66–1·04) [1869/2257]	0·037 (0·023–0·058) [434/11 852]	0·030 (0·019–0·046) [185/6244]	683	1 (ref)	1 (ref)	492	1 (ref)	1 (ref)
	High resistance (mortality <78·57%)		0·40 (0·32–0·50) [403/1001]	1·41 (1·13–1·75) [979/696]	0·82 (0·63–1·07) [2911/3541]	0·031 (0·023–0·042) [888/28 731]	0·030 (0·015–0·061) [69/2311]	683	0·93 (0·73–1·18); p=0·557	0·89 (0·72–1·10); p=0·298	492	0·97 (0·74–1·27); p=0·828	0·91 (0·73–1·14); p=0·399
	Effect per 10% reduction in bioassay mortality		..	..	..	..	..	683	0·95 (0·95–1·00); p=0·056	0·94 (0·89–0·99); p=0·018	492	0·95 (0·89–1·02); p=0·132	0·93 (0·88–0·99); p=0·029
	Effect per 10% reduction in bioassay mortality after removal of outliers		..	..	..	..	..	679	0·95 (0·90–1·0); p=0·064	0·95 (0·89–1·02) p=0·151	488	0·94 (0·89–0·99); p=0·031	0·94 (0·88–1·00); p=0·0475
Stratifying by resistance (interaction)													
	In low resistance areas (mortality ≥78·57%)												
		Non-net use	0·74 (0·52–1·06) [67/91]	..	1·60 (1·09–2·35) [114/71]	0·031 (0·019–0·052) [137/4414]	0·099 (0·056–0·177) [70/707]	307	1 (ref)	1 (ref)	251	1 (ref)	1 (ref)
		Net use	0·61 (0·44–0·84) [245/405]	..	0·80 (0·36–0·70) [1698/2120]	0·040 (0·024–0·066) [297/7438]	0·021 (0·014–0·032) [115/5354]	307	0·59 (0·37–0·94); p=0·027	0·59 (0·38–0·90); p=0·015	251	0·45 (0·27–0·74); p=0·002	0·44 (0·27–0·72); p=0·001
In high resistance areas (mortality <78·57%)													
		Non-net users	0·61 (0·39–0·96) [25/41]	..	1·29 (0·85–1·97) [225/174]	0·022 (0·015–0·031) [247/11 434]	0·068 (0·031–0·150) [19/281]	281	1 (ref)	1 (ref)	146	1 (ref)	1 (ref)
		Net users	0·39 (0·32–0·49) [378/960]	..	0·79 (0·60–1·04) [2633/3322]	0·037 (0·027–0·052) [641/17 297]	0·025 (0·011–0·055) [50/1964]	281	0·62 (0·94–10·04); p=0·068	0·51 (0·27–0·98); p=0·042	146	0·43 (0·27–0·67); p<0·0001	0·35 (0·20–0·61); p<0·0001
	Interaction parameter (net use by high resistance)		..	..	..	..	..	..	0·95 (0·48–1·90); p=0·942	1·15 (0·53–2·47); p=0·860	..	1·04 (0·53–2·07); p=0·947	1·28 (0·61–2·67); p=0·737

* Absolute numbers for incidence (cases/child-years) are given in square brackets.

† Excluding Cameroon for comparisons involving net use.

‡ Use of nets was an inclusion criterion for the cohort study in Cameroon, so the data do not provide a comparison of net users versus non-net users.

§ Adjusted for age, district, calendar month, and, in the case of Sudan, indoor residual spraying.

We found no evidence that clinical incidence differed between high and low resistance clusters for all locations and timepoints combined (table 3). We found evidence of a negative linear association between incidence and mosquito mortality (table 3; figure). After removing four outliers (defined as points whose standardised values were >4 SDs above the mean value) this association was not significant (table 3).

**Figure:**
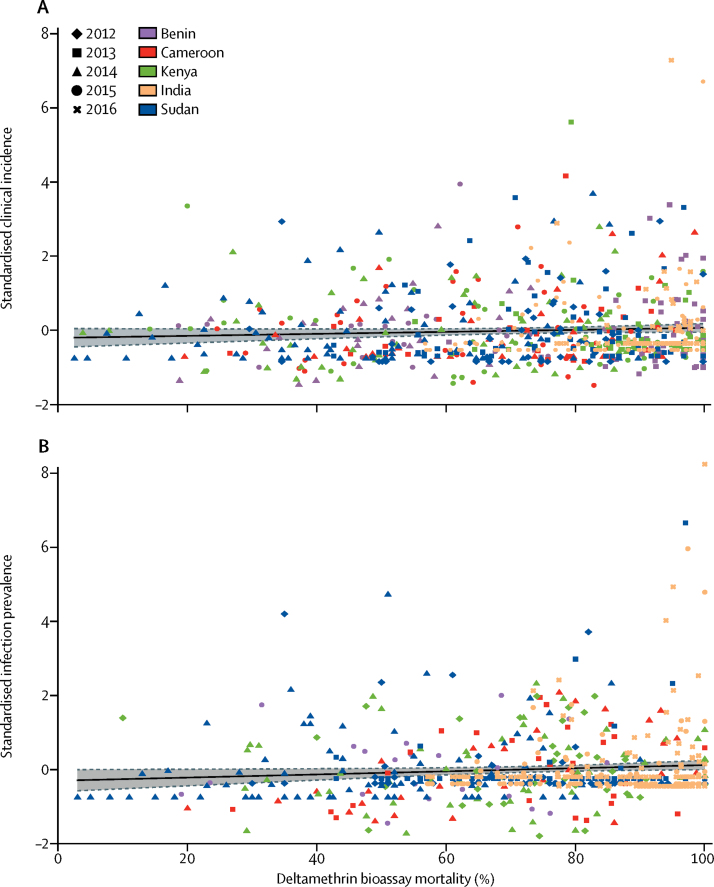
Association between mosquito mortality and malaria burden at the cluster level (A) Clinical malaria incidence and (B) prevalence of malaria infection. The line represents the regression line and the shaded area is the 95% CI.

Net users had a significantly lower risk of infection for all countries combined (table 4). However, for Sudan we found little evidence of a protective effect of net use (table 4; appendix p 7). Stratifying clusters into high and low resistance showed nearly identical effectiveness of nets across the two strata, and this result was similar when excluding Sudan (table 4).

**Table 4:** Prevalence of malaria infection

			Mean malaria infection prevalence (95% CI)*					**Effect of net use and resistance in all five countries combined**			**Effect of net use and resistance in Benin, Cameroon, India, and Kenya combined**		
			Benin	Cameroon	Kenya	India	Sudan	Total resistance observations	Unadjusted odds ratio (95% CI)	Adjusted† odds ratio (95% CI)	Total resistance observations	Unadjusted odds ratio (95% CI)	Adjusted† odds ratio (95% CI)
Overall prevalence			51·6% (45–58) [836/1621]	30·4% (25–37) [1547/5081]	34·9% (31–39) [2299/6596]	0·9% (0·5–1·5) [163/18 054]	1·9% (1·3–2·8) [367/19 348]	..	..	..	..	..	..
Caregiver-reported use of net on the previous night													
	Non-net use		54·1% (44–64) [211/390]	35·0% (24–47) [243/694]	47·0% (41–53) [132/281]	1·8% (0·9–3·9) [41/2233]	1·3% (0·7–2·1) [45/3588]	602	1 (ref)	1 (ref)	411	1 (ref)	1 (ref)
	Net use		50·8% (43–58) [625/1231]	28·6% (23–35) [798/2795]	34·4% (31–38) [2072/6021]	0·8% (0·5–1·3) [122/15 821]	2·0% (1·4–3·0) [322/15 760]	602	0·64 (0·51–0·79); p<0·0001	0·63 (0·51–0·78); p<0·0001	411	0·59 (0·46–0·76); p<0·0001	0·60 (0·47–0·78); p<0·0001
Insecticide resistance													
	Low resistance (mortality ≥78·57%)		65·1% (12–99) [97/149]	33·0% (26–41) [768/2330]	34·7% (30–39) [726/2091]	0·9% (0·5–1·7) [129/13 777]	1·8% (0·7–4·2) [99/5501]	602	1 (ref)	1 (ref)	411	1 (ref)	1 (ref)
	High resistance (mortality <78·57%)		50·2% (43–57) [739/1472]	28·3% (21–37) [779/2751]	34·9% (30–40) [1573/4505]	0·8% (0·3–2·4) [34/4277]	1·9% (1·4–2·7) [268/13 847]	602	0·83 (0·59–1·15); p=0·261	0·86 (0·70–1·06); p=0·148	411	0·88 (0·62–1·24); p=0·465	0·84 (0·68–1·05); p=0·130
	OR† per 10% reduction in bioassay mortality		..	..	..	..	..	602	0·92 (0·83–1·02); p=0·111	0·92 (0·86–0·98); p=0·015	411	0·92 (0·81–1·03); p=0·145	0·91 (0·83–1·00); p=0·043
	OR† per 10% reduction in bioassay mortality after removal of outliers		..	..	..	..	..	594	0·93 (0·85–1·01); p=0·10	0·94 (0·84–1·04); p=0·223	406	0·94 (0·87–1·01); p=0·087	0·93 (0·85–1·03); p=0·158
Stratifying by resistance (interaction)													
	In lower resistance areas (mortality ≥78·57%)												
		Non-net use	69·2% (18–96) [36/52]	36·3% (21–55) [141/388]	45·7% (37–55) [43/94]	1·9% (0·7–4·7) [31/1667]	1·3% (0·5–3·3) [19/1414]	299	1 (ref)	1 (ref)	243	1 (ref)	1 (ref)
		Net use	62·9% (2–99) [61/97]	30·5% (23–40) [396/1297]	34·3% (30–39) [635/1851]	0·8% (0·5–1·4) [98/12 110]	2·0% (0·7–5·1) [80/4087]	299	0·64 (0·44–0·91); p=0·013	0·65 (0·46–0·91); p=0·014	243	0·63 (0·43–0·94); p=0·022	0·64 (0·43–0·95); p=0·027
	In high resistance areas (mortality <78·57%)												
		Non-net users	51·8% (41–62) [175/338]	33·3% (21–48) [102/306]	47·6% (40–56) [89/187]	1·8% (0·6–4·9) [10/556]	1·2% (0·6–2·3) [26/2174]	303	1 (ref)	1 (ref)	168	1 (ref)	1 (ref)
		Net users	49·7% (42–57) [564/1134]	26·8% (20–35) [402/1498]	34·5% (30–40) [1437/4170]	0·6% (0·2–2·2) [24/3711]	2·1% (1·5–2·9) [242/11 673]	303	0·63 (0·51–0·79); p<0·0001	0·66 (0·53–0·82); p=0·0002	168	0·59 (0·46–0·77); p<0·0001	0·65 (0·50–0·83); p=0·0007
	Interaction parameter (net use by high resistance)		..	..	..	..	..	..	1·00 (0·66–1·52); p=0·992	0·98 (0·65–1·48); p=0·961	..	1·07 (0·67–1·71); p=0·890	0·99 (0·62–1·58); p=0·990

* Absolute numbers for prevalence (cases/child-years) are given in square brackets.

† Adjusted for age, district and, in the case of Sudan, indoor residual spraying.

In separate analyses, restricting the analysis to cluster-time instances in which mosquito mortality was less then 50%, we found evidence that nets provided protection against infection, but there was no evidence that they provide protection against clinical malaria (appendix pp 10–13).

We noted no association between human infection prevalence and lower mosquito bioassay mortality. Prevalence declined with decreasing mosquito mortality when resistance was modelled as a continuous variable (table 4; figure). After removing eight outliers (as defined previously) we found no evidence for an association between prevalence and mosquito mortality (table 4).

For Indian incidence data, the RR for net use without adjusting for socioeconomic status was 0·31 (95% CI 0·22–42, p<0·0001), and the RR for net use adjusted for socioeconomic status was 0·30 (0·22–0·42, p<0·0001). For prevalence, the OR before adjusting for socioeconomic status (0·35, 0·23–0·53, p<0·0001) was similar to that after adjusting for socioeconomic status (0·35, 0·23–0·52, p<0·0001). The mean difference in age between net users and non-users, adjusted for country, was 0·04 years (p=0·170).

## Discussion

Our study found that long-lasting insecticidal nets provided protection against malaria in all study countries except Sudan, despite vector resistance. We found no evidence that the amount of protection provided by long-lasting insecticidal nets differed by the frequency of resistance as measured by WHO bioassays. Similarly, we found no evidence of an association between infection prevalence or clinical incidence with higher pyrethroid resistance.

A Cochrane review^17^ of trials that preceded widespread emergence of pyrethroid resistance provided overall estimates of effectiveness of insecticide-treated nets that were similar to the estimates of effectiveness made in our study (13% for parasite prevalence and 50% for uncomplicated malaria incidence). However, several factors should be taken into account when comparing our results with those of the Cochrane review. First, our study did not test if protection provided before the emergence of resistance was the same as that observed after resistance emerged. Second, the measure of effectiveness in our study was based on comparing net users with non-users, whereas trials included in the Cochrane review compared communities that were randomised to receive nets or not. Third, the trials included in the Cochrane review were conducted at a time when most settings had higher intensity of malaria transmission than those encountered in our study settings, which is likely to affect observed effectiveness.

A 2014 meta-analysis^18^ found that treated nets offered greater protection than did untreated nets, even when vectors were resistant to pyrethroids. Another study^19^ showed that oocyst development was slower in deltamethrin-exposed plasmodium-infected resistant mosquitoes compared with unexposed individuals. A further study^20^ found delayed mortality despite initial survival beyond 24 h following exposure to insecticide on long-lasting insecticidal nets in highly pyrethroid-resistant malaria vectors. Therefore, plausible mechanisms are present through which long-lasting insecticidal nets might continue to provide at least partial protection against malaria, despite vector resistance.^21^, ^22^

The average daily survival of mosquitoes encountering treated nets should be substantially reduced with high long-lasting insecticidal net coverage and susceptible mosquitoes, resulting in a mass effect of long-lasting insecticidal nets reducing the risk of infective bites for users and non-users alike.^23^ If mosquitoes are resistant to pyrethroids, the contact irritancy and physical barrier of long-lasting insecticidal nets is likely to provide users with some protection against resistant mosquitoes seeking a blood meal,^24^ but the mass effect could diminish. Because of diversion, the risk of infection for non-users of long-lasting insecticidal nets could increase, whereas the risk for users could remain relatively unchanged. Measuring the community effect of long-lasting insecticidal nets on malaria transmission would require random assignment of communities to receive or not receive nets, which is unethical in malaria-endemic areas. However, infection rates were higher in non-users than users, even in communities with high proportions of reported net use, suggesting that nets might have provided no community protection. Pyrethroid resistance might have reduced the mass effect of long-lasting insecticidal nets, even if there was little or no measurable effect on personal protection.

Our study has several limitations. Insecticide resistance measurement was based on the WHO insecticide susceptibility test using a diagnostic dose, which measures the frequency of resistant individuals in the mosquito population, but not the intensity or strength of resistance in those individuals. Measures of resistance intensity based on a dose-response relationship^25^ would have been more informative, but for our study the standard WHO bioassay test was considered best suited for the scale and variety of settings in which insecticide susceptibility status had to be assessed. A reduction in vector control effectiveness that was not detected might have occurred in this evaluation. Resistance to pyrethroids (by WHO definition mortality <90%^13^) was observed in 78% of study clusters. Therefore, resistance might have an impact on malaria prevalence and incidence across all study clusters, but the paucity of susceptible mosquito populations (ie, ≥98% mortality) might have rendered any impact undetectable. Conversely, resistance intensity might not have reached a level that resulted in a detectable epidemiological effect. However, post-hoc analysis restricted to clusters with less than 50% bioassay mortality showed that long-lasting insecticidal nets still offered protection from malaria infection.

Because it is not possible to randomise locations to insecticide resistance or ethical to randomise children to not use nets, our study had to rely on an observational design, rendering it subject to confounding factors. In Sudan, we observed higher clinical incidence with net use than non-net use. This association is more likely to be due to those who are at higher risk of mosquito bites being more likely to use nets,^1^ rather than the implausible explanation that use of nets increased the risk of malaria infection in this setting. The observed protective effect of long-lasting insecticidal nets in the other four countries could have been exaggerated because of socioeconomic differences between users and non-users.^26^ The scale of our study precluded measurement of socioeconomic status for all children. However, in Benin^27^ and India where socioeconomic status was measured, the protective effect of nets persisted after adjusting for this factor.

Our large multicountry study found that pyrethroid long-lasting insecticidal nets protect against malaria despite vector resistance.^28^ Whatever the explanation, our results provide evidence to suggest that, regardless of insecticide resistance, populations living in malaria-endemic areas should continue to use long-lasting insecticidal nets, as this will reduce their chances of malaria infection and disease. Nevertheless, standard nets provide only partial protection against malaria, and therefore the integration of novel technologies and approaches, and new tools including new generation nets, will be essential to achieve the targets set out in the Global Technical Strategy for Malaria 2016–2030.^29^ Resistance of malaria vectors to insecticides—particularly pyrethroids—is increasing in intensity and geographical spread,^3^ and is more pronounced in countries other than where our study sites were located.^30^ Entomological data suggest reduced bednet efficacy in these locations with high resistance intensity.^31^ Resources should be mobilised to enable countries to develop and implement insecticide resistance management plans^9^ and to support development of additional vector control strategies. In the interim, efforts should be made to increase access to good condition long-lasting insecticidal nets. Use of pyrethroid nets will continue to save lives until more effective malaria prevention methods are available.
