# Supplementary material for: Implications of insecticide resistance for malaria vector control with long-lasting insecticidal nets: a WHO-coordinated, prospective, international, observational cohort study
Source: Lancet Infect Dis. 2018 Jun;18(6):640–9. doi: 10.1016/S1473-3099(18)30172-5 (PMC5968369; doi:10.1016/S1473-3099(18)30172-5)

THE LANCET Infectious Diseases

Supplementary appendix

This appendix formed part of the original submission and has been peer reviewed. We post it as supplied by the authors.

Supplement to: Kleinschmidt I, Bradley J, Kno TB, et al. Implications of insecticide resistance for malaria vector control with long-lasting insecticidal nets: a WHO-coordinated, prospective, international, observational cohort study. *Lancet Infect Dis* 2018; published online April 9. http://dx.doi.org/10.1016/S1473-3099(18)30172-5.

APPENDIX

Table A1: Cluster level sample sizes and variation of outcome measure from cross sectional surveys

Country	Year of survey	Median (interquartile range) of cluster level sample size	Intracluster correlation in malaria prevalence	Median (interquartile range) of cluster level malaria prevalence (%)
**Benin**	2015	78 (71 - 83)	0.10	51.9 (40.5 – 58.9)
**Cameroon**	2013	81 (80 - 86)	0.21	30.5 (10.2 – 48.8)
	2014	82 (80 - 84)	0.10	27.5 (15.5 – 43.9)
**Kenya**	2012	38 (27 - 47)	0.07	26.1 (20.3 – 37.3)
	2014	103 (84 - 115)	0.09	34.7 (26.9 – 45.2)
**India**	2015	76 (63 84)	0.48	0.0 (0.0 – 1.3)
	2016 (June)	80 (67-88)	0.51	0.0 (0.0 – 0.0)
	2016 (November)	80 (67-88)	0.22	0.0 (0.0 – 1.2)
**Sudan**	2012	98 (94 - 101)	0.48	0.0 (0.0 – 1.0)
	2013	92 (74 - 102)	0.51	0.0 (0.0 – 0.0)
	2014	100 (97 – 140)	0.33	1.1 (0.0 – 3.2)

Table A2: Cluster level sample sizes and variation of outcome measure from study cohorts

Country	Year of cohort	Median (interquartile range) of cluster level number of children in the cohort	Median (interquartile range) of cluster level child years follow up	Coefficient of variation of malaria incidence	Median (interquartile range) of cluster level malaria incidence (cases per child per year)
Benin	2013 (Jan - Jul)	30 (29 - 31)	11.2 (9.2 – 11.7)	0.86	0.47 (0.25 – 0.87)
	2013 (Aug - Dec)	28 (26 -29)	8.1 (7.5 – 8.7)	0.86	0.54 (0.35 – 0.94)
	2014	28 (26 - 29)	22.9 (21.4 – 24.2)	0.63	0.54 (0.24 – 0.75)
	2015	50 (48 - 50)	20.7 (18.7 – 21.8)	1.02	0.20 (0.09 -0.23)
Cameroon	2013	60 (57 - 60)	13.9 (11.5 – 15.3)	1.21	1.35 (0.41 – 2.39)
	2014	60 (58 - 60)	11.6 (9.6 – 12.9)	1.42	0.18 (0.00 – 0.79)
	2015	60 (60 - 60)	11.5 (10.1 – 13.1)	0.68	2.01 (1.13 – 3.24)
Kenya	2013	80 (80 - 81)	5.0 (2.2 – 10.6)	3.01	0.62 (0.24 – 2.17)
	2014	83 (80 - 86)	64.2 (57.2 – 67.7)	0.74	0.81 (0.29 – 1.19)
	2015	80 (80 - 84)	59.9 (55.0 – 62.9)	0.71	0.64 (0.39 – 1.14)
India	2015	86 (71 - 95)	86 (73 - 96)	1.90	0.01 (0.00 - 0.05)
	2016	85 (71 - 95)	24.3 (20.0 – 27.4)	2.89	0.00 (0.00 – 0.03)
Sudan	2012	238 (228- 251)	196.4 (191.4 - 200.0)	1.19	0.02 (0.00 – 0.06)
	2013	241 (235 - 254)	219.9 (211.8 – 226.2)	1.46	0.01 (0.04 – 0.03)
	2014	249 (240 - 260)	218 (213 - 226)	1.33	0.01 (0.00 – 0.04)

Table A3: LLIN use in children under 5 years from cross-sectional surveys, by country and year

Country	Year of survey	Net use the night before survey, % (n/N)
**Benin**	2015	78% (694/895)
**Cameroon**	2013	87% (824/950)
	2014	74% (641/862)
**Kenya**	2012	92% (1381/1508)
	2014	97% (4639/4793)
**India**	2015	95% (1985/2092)
	2016 (June)	82% (1816/2202)
	2016 (November)	90% (2000/2224)
**Sudan**	2012	84% (1866/2232)
	2013	66% (1596/2406)
	2014	92% (2562/2796)

Figure A1: Estimated rate ratios for personal protection provided by nets on clinical malaria incidence.

(A) In all study clusters (B) In low resistance study clusters (mosquito mortality >= 78.57%) (C) In high resistance study clusters (mosquito mortality < 78.57%). On each graph the red dashed line is the overall rate ratio and the grey boxes are proportional to the random effects study weights. Estimates are adjusted for age, district, calendar month, and, in the case of Sudan, indoor residual spraying.

(A)

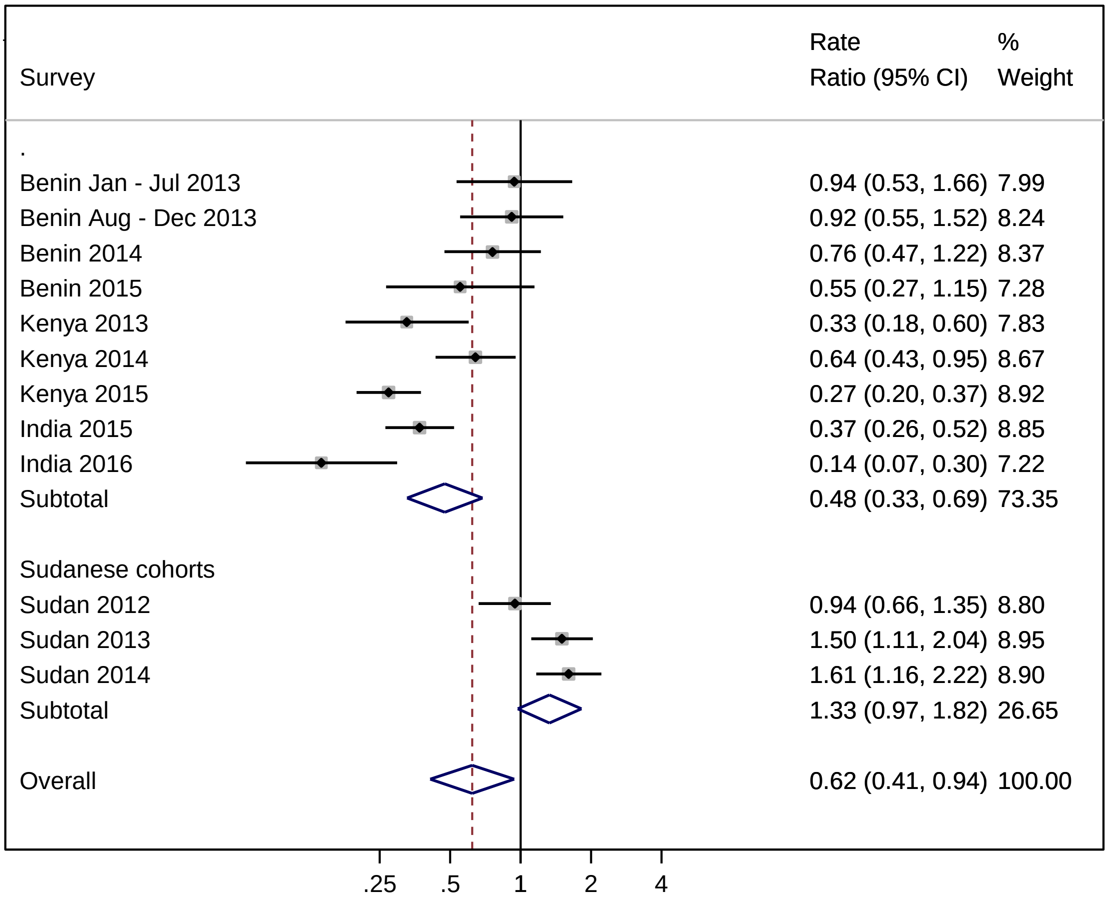

(B)

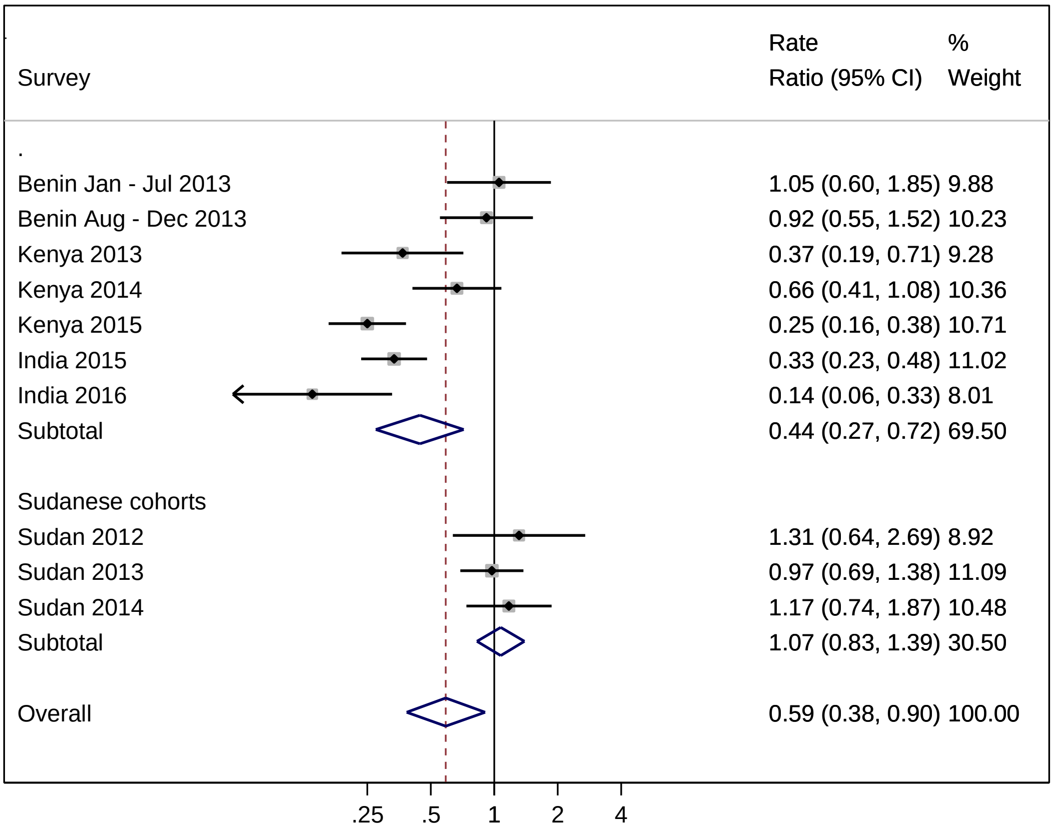

Survey
Rate Ratio (95% CI)
% Weight
.
Benin Jan - Jul 2013 1.05 (0.60, 1.85) 9.88
Benin Aug - Dec 2013 0.92 (0.55, 1.52) 10.23
Kenya 2013 0.37 (0.19, 0.71) 9.28
Kenya 2014 0.66 (0.41, 1.08) 10.36
Kenya 2015 0.25 (0.16, 0.38) 10.71
India 2015 0.33 (0.23, 0.48) 11.02
India 2016 0.14 (0.06, 0.33) 8.01
Subtotal 0.44 (0.27, 0.72) 69.50
Sudanese cohorts
Sudan 2012 1.31 (0.64, 2.69) 8.92
Sudan 2013 0.97 (0.69, 1.38) 11.09
Sudan 2014 1.17 (0.74, 1.87) 10.48
Subtotal 1.07 (0.83, 1.39) 30.50
Overall 0.59 (0.38, 0.90) 100.00
.25
.5
1
2
4

(C)

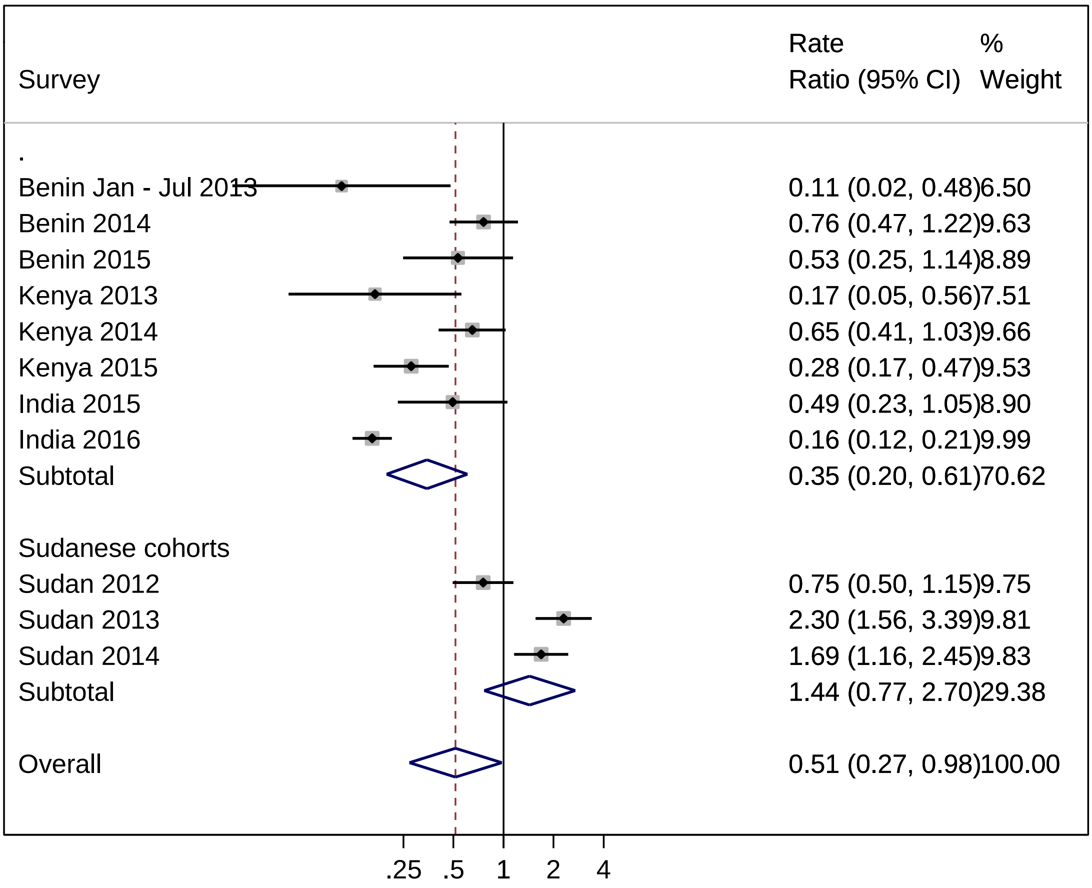
Survey
Rate Ratio (95% CI)
% Weight
.
Benin Jan - Jul 2013
Benin 2014
Benin 2015
Kenya 2013
Kenya 2014
Kenya 2015
India 2015
India 2016
Subtotal
Sudanese cohorts
Sudan 2012
Sudan 2013
Sudan 2014
Subtotal
Overall
0.11 (0.02, 0.48)6.50
0.76 (0.47, 1.22)9.63
0.53 (0.25, 1.14)8.89
0.17 (0.05, 0.56)7.51
0.65 (0.41, 1.03)9.66
0.28 (0.17, 0.47)9.53
0.49 (0.23, 1.05)8.90
0.16 (0.12, 0.21)9.99
0.35 (0.20, 0.61)70.62
0.75 (0.50, 1.15)9.75
2.30 (1.56, 3.39)9.81
1.69 (1.16, 2.45)9.83
1.44 (0.77, 2.70)29.38
0.51 (0.27, 0.98)100.00
.25
.5
1
2
4

Figure A2: Estimated odds ratios for personal protection provided by nets on malaria infection prevalence.

(A) In all study clusters (B) In low resistance study clusters (mosquito mortality >= 78.57%) (C) In high resistance study clusters (mosquito mortality < 78.57%). On each graph the red dashed line is the overall odds ratio and the grey boxes are proportional to the random effects study weights. Estimates are adjusted for age, district and, in the case of Sudan, indoor residual spraying.

(A)

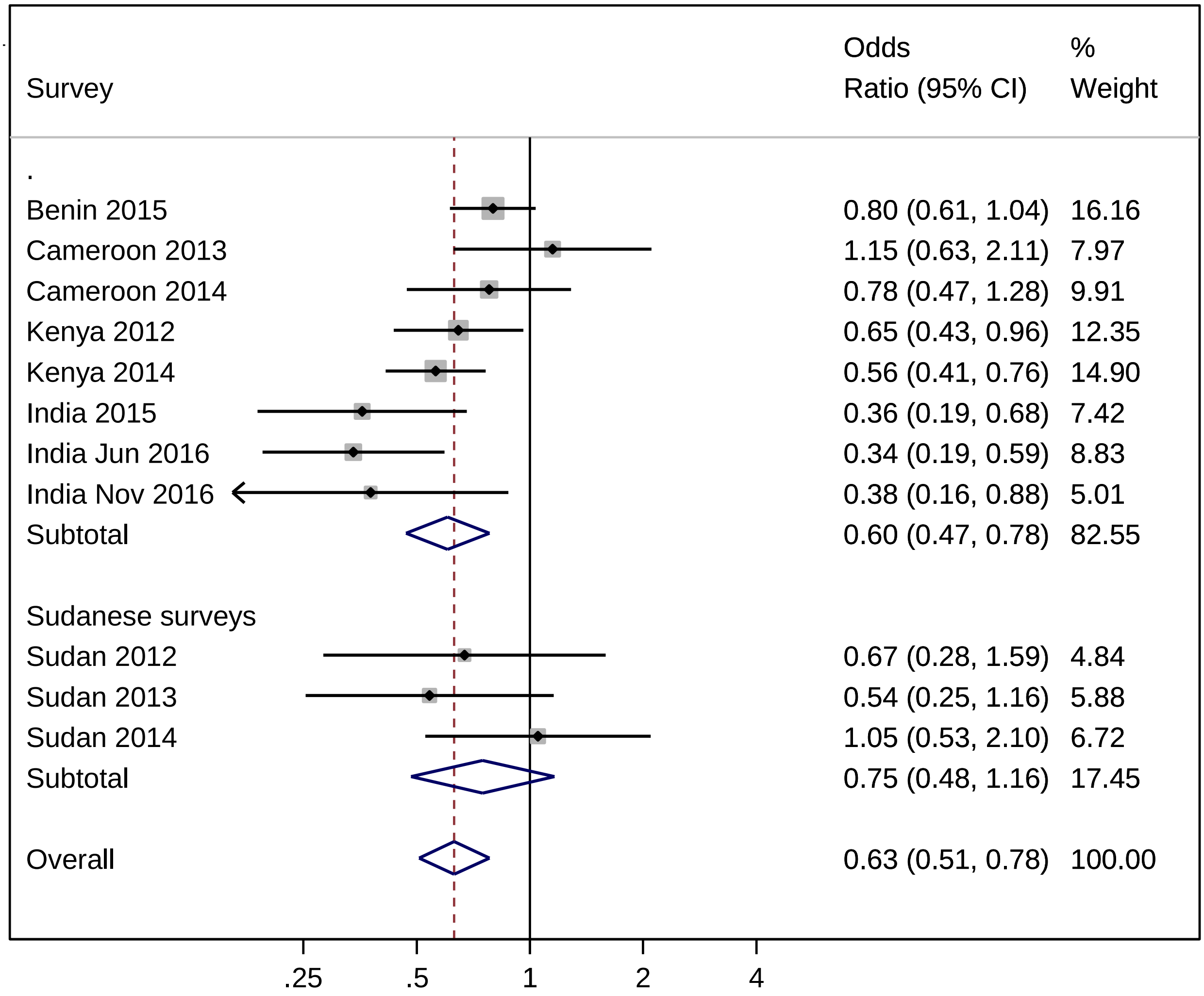

(B)

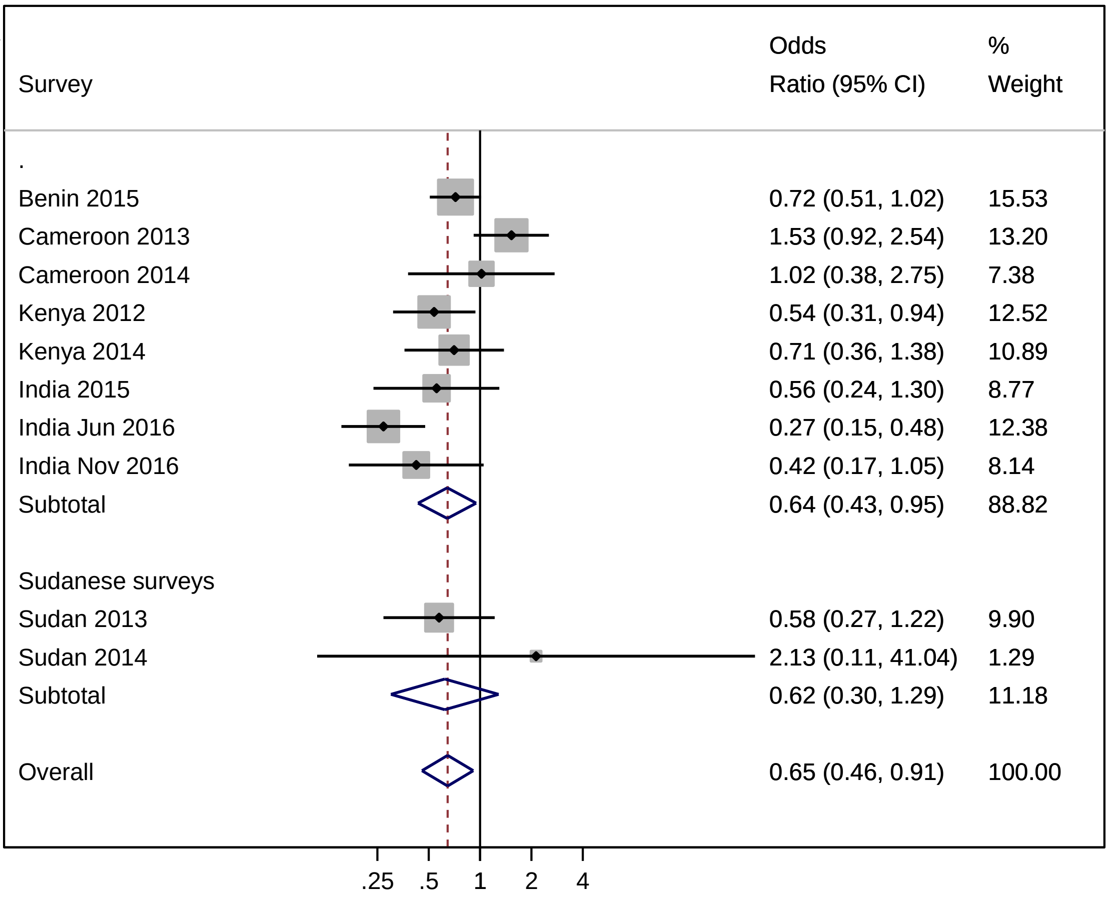
Survey
Odds Ratio (95% CI)
% Weight
Benin 2015 0.72 (0.51, 1.02) 15.53
Cameroon 2013 1.53 (0.92, 2.54) 13.20
Cameroon 2014 1.02 (0.38, 2.75) 7.38
Kenya 2012 0.54 (0.31, 0.94) 12.52
Kenya 2014 0.71 (0.36, 1.38) 10.89
India 2015 0.56 (0.24, 1.30) 8.77
India Jun 2016 0.27 (0.15, 0.48) 12.38
India Nov 2016 0.42 (0.17, 1.05) 8.14
Subtotal 0.64 (0.43, 0.95) 88.82
Sudanese surveys
Sudan 2013 0.58 (0.27, 1.22) 9.90
Sudan 2014 2.13 (0.11, 41.04) 1.29
Subtotal 0.62 (0.30, 1.29) 11.18
Overall 0.65 (0.46, 0.91) 100.00
.25
.5
1
2
4

(C)

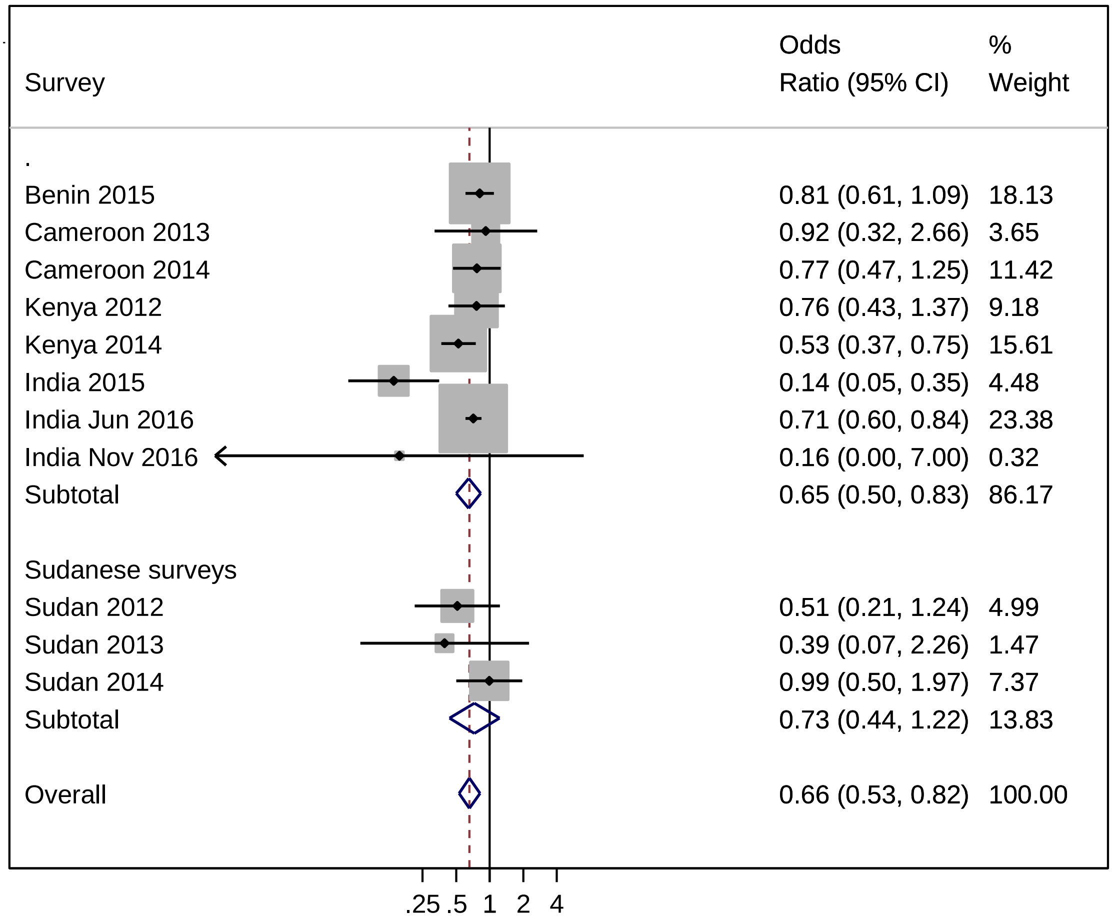

Survey
Odds
Ratio (95% CI)
%
Weight
.
Benin 2015
0.81 (0.61, 1.09)
18.13
Cameroon 2013
0.92 (0.32, 2.66)
3.65
Cameroon 2014
0.77 (0.47, 1.25)
11.42
Kenya 2012
0.76 (0.43, 1.37)
9.18
Kenya 2014
0.53 (0.37, 0.75)
15.61
India 2015
0.14 (0.05, 0.35)
4.48
India Jun 2016
0.71 (0.60, 0.84)
23.38
India Nov 2016
0.16 (0.00, 7.00)
0.32
Subtotal
0.65 (0.50, 0.83)
86.17
Sudanese surveys
Sudan 2012
0.51 (0.21, 1.24)
4.99
Sudan 2013
0.39 (0.07, 2.26)
1.47
Sudan 2014
0.99 (0.50, 1.97)
7.37
Subtotal
0.73 (0.44, 1.22)
13.83
Overall
0.66 (0.53, 0.82)
100.00
.25
.5
1
2
4

Table A4: Association between nets and malaria infection in clusters with mosquito mortality < 50%

Country	Number of clusters	Reported use of net the previous night	Malaria prevalence, % [95% CI] (N)
Benin	6	No	62% [25, 89] (97)
		Yes	53% [0.35, 0.70] (396)
Cameroon	8	No	19% [15, 24] (114)
		Yes	8.4% [5.7, 12.2] (417)
Kenya	13	No	48% [35, 61] (71)
		Yes	35% [27, 44] (1265)
Sudan	38	No	2.8% [1.1, 6.8] (528)
		Yes	2.0% [1.2, 3.5] (3638)

Figure A3: Estimated odds ratios for personal protection provided by nets on malaria infection prevalence in study clusters with mosquito mortality < 50%

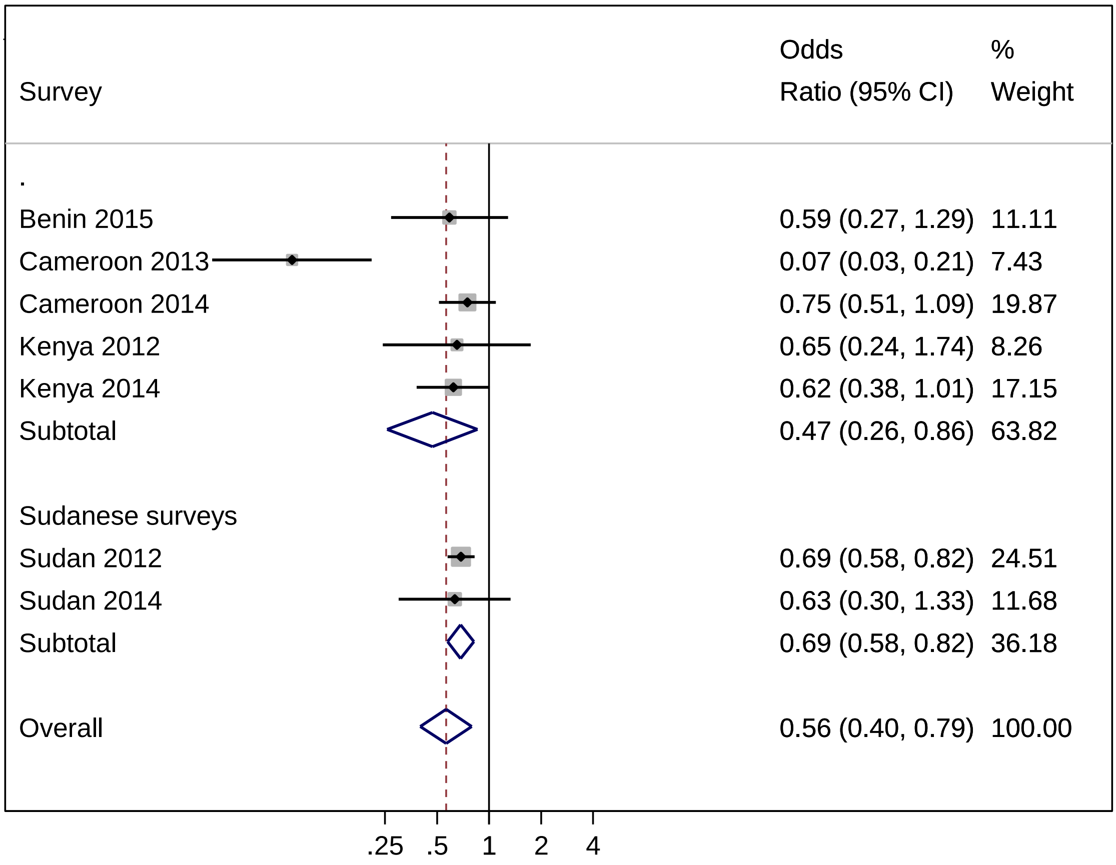

Table A5: Association between nets and clinical malaria incidence in clusters with mosquito mortality < 50%

Country	Number of clusters	Reported use of net the previous night	Malaria incidence, rate per child year (follow up time)
Benin	14	No	0.57 [0.26, 1.25] (12)
		Yes	0.40 [0.29, 0.55] (313)
Kenya	19	No	1.13 [0.57, 2.24] (71)
		Yes	0.80 [0.55, 1.15] (1654)
Sudan	38	No	0.017 [0.011, 0.027] (3809)
		Yes	0.034 [0.021, 0.054] (4786)

Figure A4: Estimated rate ratios for personal protection provided by nets on clinical malaria incidence in study clusters with mosquito mortality < 50%.

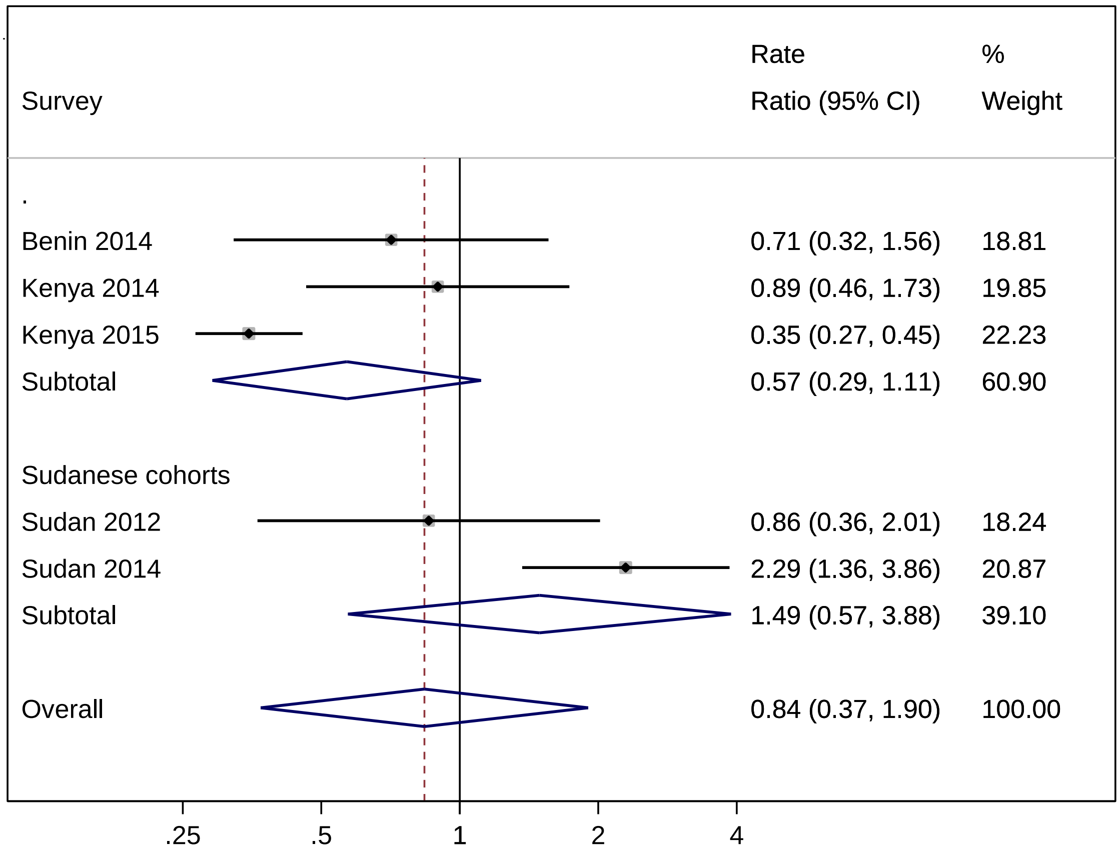

Supplement: Supplementary appendix [file mmc1.pdf]
